# Reconsidering Routine DNR Reversal for Interventional Cardiac Procedures

**DOI:** 10.1016/j.jacadv.2025.102115

**Published:** 2025-09-17

**Authors:** Kaitlyn A. Stoehr, Jay R. Malone

**Affiliations:** a Yale School of Medicine, New Haven, Connecticut, USA; b Department of Pediatrics, Washington University School of Medicine, St. Louis, Missouri, USA

**Keywords:** autonomy, cardiac catheterization, code status, do not resuscitate (DNR), geriatric cardiology, medical ethics, percutaneous coronary intervention (PCI)

Tom, an 84-year-old, has multivessel coronary artery disease and recently underwent percutaneous coronary intervention (PCI) for a non–ST-segment elevation myocardial infarction. He now presents to the emergency department with recurrent chest pain and is again diagnosed with non–ST-segment elevation myocardial infarction, likely from stent thrombosis. The interventional cardiologist, Dr Atria, discusses repeat PCI and Tom consents.

Dr Atria notes that Tom has an active do not resuscitate (DNR) order and informs him of the hospital’s policy: all DNR orders are suspended during and for 24 hours after interventional cardiac procedures. Tom objects, recalling his brother’s prolonged intensive care unit stay: “I’d rather go quickly than wake up on a ventilator.”

Caught between institutional policy and Tom’s clearly expressed values, Dr Atria hesitates. Should she proceed with PCI while respecting his DNR? Can she ask him to reconsider? What does it mean to honor autonomy in a setting designed to override physiological failure?

DNR status during and immediately after cardiac interventions carries significant implications for patient care; however, professional guidelines provide limited direction beyond endorsing reversal. In 2016, the Society for Cardiovascular Angiography and Interventions recommended automatic suspension of DNR orders:

“[During informed consent] specific mention should be made of do not resuscitate (DNR) status and that it has been revoked for the duration of the procedure and a minimum of 24 hr following the procedure, with the interventional cardiologist involved in any decision to reinstate DNR.”^1^

A 2021 update reinforced this presumption:

“It is important to discuss temporary suspension [of the DNR] with the patient or health care proxy and document the terms and duration of the temporary suspension.”^2^

These statements reflect a default expectation of DNR suspension while offering little guidance to clinicians navigating the decision-making process. Empirical data on institutional practices are limited. A study of transcatheter aortic valve replacement programs revealed considerable policy variation, including inconsistent requirements for DNR reversal and differing reinstatement time frames.^3^

Code status is particularly pertinent in cardiac interventions, where procedural manipulation can lead to sudden, but sometimes readily reversible, complications. At the same time, patients undergoing these procedures, who are often older and have multiple comorbidities, face elevated baseline risks of spontaneous cardiac arrest that may be unrelated to the intervention itself. For patients with DNR orders, this convergence of procedural and nonprocedural risk makes preprocedural code discussions ethically urgent.

Hospitals are required to inform patients of their right to refuse care and to establish advance directives, including DNR orders.^4^ These protections stem from the principle of autonomy, which is an extension of respect for persons. A DNR order allows patients to articulate specific limits on life-sustaining interventions and assert control over how they approach the end of life. Crucially, a DNR does not constitute broader refusal of care; it applies only in the event of cardiac or respiratory arrest. Tom’s case illustrates that agreeing to a procedure does not necessarily imply reconsideration of the values that initially prompted a DNR. Yet patients may not have contemplated the periprocedural context when initially establishing their preferences, raising the question: Does a new procedural risk environment justify default DNR suspension?

Two arguments are often presented favoring procedural DNR suspension. First, some note that the methods for restoring vital functions after general anesthesia are indistinguishable from what would be termed “resuscitation” in other settings. Second, because procedural arrests are often iatrogenic, some contend that clinicians have a stronger obligation to attempt resuscitation. Both arguments seem to favor DNR suspension. We argue that the periprocedural setting is ethically distinct from general medical care, but this distinction does not justify a blanket reversal of DNR orders. Instead, we advocate for an individualized, values-based approach to periprocedural code status.

To the first argument: if Tom refuses standard resuscitation from anesthesia on the grounds of his DNR and Dr Atria cannot find a safe alternative, they face an impasse. In practice, however, this concern is likely theoretical; Tom’s priorities suggest he is unlikely to reject an integral component of PCI, namely anesthesia. During the informed consent process, both induction and recovery from anesthesia must be explained. If Tom sees no conflict with his DNR, we should not infer one. If he does, then clarifying his concerns, as during any DNR conversation, may help alleviate them.

To the second argument: we acknowledge a distinction between procedural and unanticipated arrests. The distinction, however, does not rely on iatrogenic vs noniatrogenic causes. For instance, we would not insist upon resuscitation for a patient dying from sepsis solely because it was caused by an iatrogenic infection rather than chronic illness.^5^ Instead, the difference lies in the fact that some intraprocedural arrests may be easily reversible without long-term sequelae. Many patients do not consider this type of resuscitation when establishing their DNR.

For capacitated adult patients, autonomy is often prioritized over beneficence regarding treatment refusal. It is well established that competent adults may decline medical care, even to their own detriment. A key ethical concern is that patients may not fully understand what their DNR would prohibit in the periprocedural context. For many patients, their DNR is not intended to reject straightforward resuscitation with likely favorable outcomes. Tom wants to avoid prolonged hospitalization and mechanical dependence; with further exploration, Dr Atria can clarify whether Tom would consider intraprocedural resuscitation if a complication were easily reversible. However, not all complications are easily reversible, and older adults also face elevated baseline risk for spontaneous arrest unrelated to procedures. Complete DNR suspension would result in resuscitation even if the cause were not easily reversible, leading to outcomes that may contradict patient goals.

Autonomous decision-making relies on the physician’s obligation to ensure a thorough informed consent process, as patients need adequate information to make informed decisions. Tom’s case highlights that neither reversing nor maintaining a DNR may fully realize patient goals. In this scenario, we can refer to the American Society of Anesthesiologists, which recommends 3 options for DNR modification during anesthesia (Table 1).^6^ We have included suggested phrasing to help clinicians navigate these conversations more effectively.

**Table 1:** Intraprocedural DNR Alternatives

DNR Approach	Explanation	Suggested Clarifying Language
1. Full attempt at resuscitation	Full DNR reversal.	“If your heart stops during the procedure, we will use all available methods to try to restart it.”
2. LAR with regard to the patient’s goals and values	Patient permits the procedural team to use clinical judgment to decide which resuscitation procedures are appropriate given their knowledge of the patient’s goals and values.	“If your heart stops because of a complication that is quickly fixable, we will try to restart your heart if we believe we can respect your goal of [*insert goal here*, eg, *avoiding a ventilator or avoiding a long ICU stay*, etc]”
3. LAR with regard to specific procedures	Patient elects to continue to refuse specific procedures (such as chest compressions or tracheal intubation), which must not be deemed essential to the success of the proposed procedure.	“If your heart stops because of a complication that is quickly reversible, we will try to help, but will respect your wish not to use [*insert intervention here*, eg, *chest compressions*.]”

Potential approaches to intraprocedural code status.^6^

DNR = do not resuscitate; ICU = intensive care unit; LAR = limited attempt at resuscitation.

After Dr Atria understands Tom’s priorities, it may be appropriate to offer a limited attempt at resuscitation to respect Tom’s values. This would permit Dr Atria to perform resuscitation if she believes a complication is readily reversible, but not if it would likely result in unwanted dependence on life-sustaining technology. This option leverages her expertise in addressing various types of intraprocedural complications, complemented by her understanding of Tom’s priorities. While the periprocedural period presents unique challenges to the DNR, the immediate availability of the physician during this time facilitates a tailored approach to code status and an opportunity to better respect patient wishes.

Translating individualized DNR planning into bedside practice requires a structured yet adaptable approach. During preprocedural consent, clinicians should begin a focused code status discussion by: 1) clarifying the patient’s understanding of their current DNR order; 2) explaining reversible procedural complications; and 3) discussing whether limited resuscitative measures would align with the patient’s goals. A helpful prompt might be: “If your heart stopped during the procedure because of a problem we could quickly fix, would trying to restart your heart in that situation be consistent with your goals?” Documenting this conversation clearly in the medical record, including specific interventions the patient accepts or declines, is essential for respecting informed preferences. Institutions might use modified DNR consent templates to promote consistency and reduce disparities in consent processes.

It is possible that, even after discussion, Tom accepts standard anesthetic practices but declines changes to his code status. Dr Atria's next step should be to clarify the specific procedures Tom wishes to decline. This approach aligns with the American Society of Anesthesiologists’ “limited attempt at resuscitation with regard to specific procedures” option (Table 1) and minimizes uncertainty about which interventions Tom considers unacceptable.

If Dr Atria is uncomfortable with Tom’s choices, can she decline the procedure? By declining, she would withhold a beneficial treatment based on Tom’s refusal of intervention on a theoretical complication. Tom’s limited code status creates elevated risk of intraoperative mortality; if Dr Atria believes the elevated risk outweighs the potential benefits of the procedure, she is justified in declining. However, she must carefully evaluate how her values influence her decision. As an interventionalist, Dr Atria’s professional identity is tied to her commitment to beneficence and nonmaleficence. Allowing a procedural death without attempting resuscitation may conflict with her core values. Dr Atria must acknowledge that Tom’s values regarding his care are distinct from her own and consider whether refusing is genuinely patient-centered or stems from personal discomfort. In some cases, declining an intervention may increase mortality more than proceeding with a DNR in place, suggesting that physician refusal reflects the difference between active and passive harms rather than patient-centered decision-making. However, clinicians may experience significant distress if they witness a preventable procedural death without intervening, particularly for iatrogenic complications with straightforward solutions. This moral burden is significant, and the potential for adverse outcomes in physician mental health deserves consideration. If Dr Atria decides not to proceed, she should consider transferring Tom’s care to another physician, provided one is available in an appropriate time frame.

Institutional practices that withhold procedures unless DNRs are reversed fail to meet the standard of individualized risk assessment. Moreover, such policies risk compelling patients to accept unwanted code status changes, especially when intervention is urgently indicated. Institutional policy variability may disproportionately impact patients from socioeconomically disadvantaged backgrounds. Ensuring that modified code status planning is accessible and clearly communicated across literacy levels is essential to equitable implementation.

It is also important to recognize that patient outcomes reporting may influence clinician decision-making or institutional policy. Public outcomes reporting may lead physicians to avoid offering high risk but necessary PCIs,^7^ and a study revealed that registry reporting requirements influenced at least one institution to mandate DNR reversal before transcatheter aortic valve replacement.^3^ DNR practices are particularly vulnerable to such influences because maintaining DNR status during a procedure increases intraprocedural mortality risk. Because individual physicians are unlikely to change institutional risk aversion, it is incumbent on the cardiology community to modify reporting practices to avoid penalizing clinicians or institutions that provide patient-centered care. Other procedural specialties, such as gastroenterology, have addressed similar challenges. Some advocate for modified code status frameworks reflecting patient values while maintaining procedural integrity.^8^ Integrating insights from other procedural fields could help develop interdisciplinary best practices for respecting patient autonomy across procedural environments.

It is essential to distinguish between intraoperative and postoperative arrests. Intraoperative events like transient arrhythmias occur in a setting with immediate access to corrective measures and can often be rapidly corrected. In contrast, postoperative arrests are more likely to reflect the patient’s underlying condition and are less likely to result in favorable outcomes. This distinction has ethical significance, as patients may reasonably choose to permit intraoperative resuscitation while refusing aggressive postprocedural intervention. Blanket DNR reversals obscure these differences, potentially leading to resuscitation contrary to patient goals.

Unless the cardiologist anticipates a concrete risk of reversible complications occurring after (and due to) the procedure, respect for patients dictates a return to the patient’s preferred code status following anesthetic recovery. The patient may choose to revisit their code status at any time, but requiring postoperative code status reversal that does not correspond with specific risks fails the patient by increasing the risk of unwanted resuscitation.

Demonstrating respect for patients with DNRs who present for interventional cardiac procedures requires a level of nuance that institutional policies cannot provide. The specific requirements of the catheterization laboratory necessitate careful consideration of DNR status. The most ethically significant distinction between the catheterization laboratory and nonprocedural settings is the elevated risk of relatively easily correctable complications, of which most patients were likely unaware when establishing their DNR. However, this does not justify mandatory DNR reversal. Instead, it underscores the imperative for thorough informed consent processes to explain these risks to patients, understand their values, and help them choose an acceptable approach. Throughout these conversations, physicians must be mindful of their own values and motivations and prioritize respect for patients. Finally, the patient-oriented approach described herein may not be fully attainable until protections are implemented in outcomes registries for institutions that enable patients to maintain DNR status during cardiac interventions. By offering greater nuance in periprocedural DNR status and advocating for supportive reporting practices, clinicians can better align decision-making with patient values in the cardiac catheterization setting.

## Funding support and author disclosures

The authors have reported that they have no relationships relevant to the contents of this paper to disclose.
